# Possession vs. goal scoring during small-sided games in soccer: a narrative mini-review on the effects on physiological, physical, technical and tactical performance

**DOI:** 10.3389/fspor.2026.1784555

**Published:** 2026-02-26

**Authors:** Michael C. Rumpf, Johannes Jäger, Rhodri Lloyd, Matthias Lochmann

**Affiliations:** 1 Department of Sport Science and Sport, Friedrich-Alexander University Erlangen-Nürnberg, Erlangen, Germany; 2 Footballscience.net, Dreieich, Germany; 3 Sport Performance Research Institute New Zealand, AUT-University, Auckland, New Zealand; 4 Youth Physical Development Center, Cardiff School of Sport and Health Sciences, Cardiff Metropolitan University, Cardiff, United Kingdom; 5 Center for Sport Science and Human Performance, Waikato Institute of Technology, Hamilton, New Zealand

**Keywords:** ball possession, coaching, football, goal scoring, training

## Abstract

Different task constraints such as possession play and goal scoring are regularly utilized in small-sided games (SSG) in order to train game specific situations. This narrative mini-review aimed to summarize how these two task constraints influence players' physiological, physical, technical and tactical parameters during SSG. Possession games increase the physiological load of players. Variables such as mean (+4.35%) and max heart rate (+6.43%) as well as duration >90%HRmax (+39.7%) were significantly higher compared to scoring format SSG. Players also felt significantly greater subjective exertion (+24.2%), mental challenge (+18.4%) and lower enjoyment (−6.45%) in comparison to scoring games, especially when goalkeepers participated. Possession games also increased physical strain for players during SSG, with total (+12.6%) and relative (+8.43%) running distance, average moving speed (+14.2%), number of low (+35.9%) and high ACC (+23.6%), and distances in high(er) speed bands (>14 km/h) being more prominent in possession games. Players are technically more engaged in possession format SSG. Significantly more total (+38.7%) and individual (+60.4%) ball touches, number of passes (+99.0%), and passes per player (+55.8%) were observed in possession compared to scoring games. The scarce scientific evidence regarding the effect of possession games on tactical variables suggests that more players are involved for a longer period of time exploring greater space during these types of games. Coaches should apply possession games to increase physiological, physical, and technical engagement of players during SSG.

## Introduction

Ball possession has been a topic of interest for coaches and sport scientists alike over a varied of different sports such as basketball (1, 2), field hockey (3), ice-hockey (4), futsal (5) and soccer (6). Generally, it was questioned how possession is connected to winning and for the latter sports multiple scientific investigations connected ball possession to successful teams (7–11) in national championships (9–13) as well as international tournaments (7, 8). On the contrary, it was also observed that ball possession did not significantly influence game outcome (14) and furthermore longer ball possession duration was even linked to losing (15) and unsuccessful teams (16). A relative recent systematic review (17) concluded that ball possession is not related to match outcome and being highly variable itself depending on seasonal variations, characteristics of the leagues (12), playing style (18) as well as other factors such as game location (home vs. away), game status (winning vs. drawing vs. losing) and opposition quality (15, 19).

Furthermore, ball possession was observed to affect the team's technical and physical performance (17, 18, 20, 21). For the technical variables teams characterized by possession-play achieved higher values in variables related to passes (21–23) and goalscoring (e.g., pass accuracy, shots inside the box) (18). However, the effect on physical performance was not as consistent and dependent on style of play (23), positional (20, 24, 25) and competition (20) circumstances. Firstly, possession playing style was physically more strenuous compared to counterattacking over the entire match (23). Scientific evidence from the English Premier League (21), the Chinese Super League (24) and from players competing in the FIFA World Cup (22) did not show significant differences in crucial variables between high- and low-possession teams. However, players in the Spanish LaLiga applying very high ball possession showed lower physical running per minute at any speed (25), while players in the UEFA Champions League employing high ball possession strategies experience greater sprinting and high-intensity running (20). Given these contradicting circumstances, ball possession remains a topic of interest, especially as it remains a common feature incorporated into small-sided games (SSG) of the training routine of modern-day teams However, no study to date has compared ball possession small-sided games SSG vs. those promoting goal scoring. This seems crucial to further inform the training and load monitoring process. Therefore, this narrative mini-review aimed to synthesize the evidence on the effects of ball possession SSG compared with goal scoring formats in male players.

## Methods

While methods for literature searches are not mandatory for narrative reviews their inclusion can help to circumvent the increased risk for selection and inclusion bias (26), associated with a narrative style of review especially, when limited number of references are present (27). Therefore, a basic search strategy was adopted, inclusive of inclusion/exclusion criteria. Major databases (PubMed, Web of Science, SportDiscus, and Scopus, Google scholar) were searched from the earliest date available until November 2025 using various combinations of the following key words “coaching”, “training”, “possession”, “soccer”, “football”, “small-sided games”, “scoring”, “goal”, “goal number”. This search was supplemented by utilizing grey literature (https://opengrey.eu) and reference list screening of included studies, along with searches within previous reviews on similar topics. Identification of suitable articles involved the initial screening of the titles and abstracts while the second stage comprised a full-text review of the remaining articles. To be included in this review, studies needed to focus on possession and goal scoring during SSG and its effect on physiological, physical, technical and tactical parameter. Strength of evidence synthesis of quality assessments were not conducted as not required (26). All included references and the effect of possession soccer on outcome variables can be observed in Table 1.

**Table 1:** All references, regarding the effect of possession SSG on outcome variables.

Reference	Population	Playing format	Protocol	Outcome in variable in possession games (vs. scoring games)
Bujalance-Moreno et la. (29)	*N* = 16, male amateur players 23.9 years, >9 years of football experience	4 vs. 4 Possession	4 × 4 min duration for each game, 2 min passive recovery	Physiological Variables: RPE ↑ (19.2%), Dur 85–89% HRmax ↓ (−48.0%)
				Physical Variables: TD↑ (13.8%), relative TD ↑ (13.8%), Ave moving speed ↑ (16.4%), Dist 7–12.9 km/h ↑ (46.2%), #high-ACC ↑ (23.6%), #low-ACC ↓ (35.9%)
		4 vs. 4 Mini-goals		
Castellano et al. (32)	*N* = 14, male semi-professional players 21.3 years, 12.5 years of soccer experience, 3–4 training session/week	3 vs. 3	6 min bout duration, 5 min passive recovery	*Game format possession games*
		5 vs. 5		Physical Variables: TD 7 vs. 7 ↑ (+4.49%) vs. 5 vs. 5 ↑ (+23.0%) vs. 3 vs. 3, work-to-rest ratio 7 vs. 7 ↑ (+18.4%) vs. 5 vs. 5 ↑ (+113%) vs. 3 vs. 3
		7 vs. 7		
				*Possession games*
		Possession, mini goals, GK		Physiological Variables: HRAve ↑ (4.84%), HRmax ↑ (2.05%)
				Physical Variables: TD↑ (17.2%), player load ↑ (21.0%), work-to-rest ratio ↑ (58.6%)
Coutinho et al. (41)	*N* = 18, elite male youth players 13.6 years, >4.9 years of experience and representative of region	4 vs. 4 Possession	5 min bout duration, 3 min passive recovery	Physical Variables: TD↑ (10.5%), Dist jogging (3.6–14.3 km/h) ↑ (16.2%), Dist walking (0.0–3.5 km/h) ↓ (−13.3%), Ave running speed ↑ (10.3%)
		4 vs. 4 Four mini-goals		Tactical Variables: Exploration index ↑ (16.2%), Dist nearest teammate ↓ (−7.99%), Dist nearest opponent ↓ (−25.2%), passing decision-making ↓ (−10%)
da Silva et al. (36)	*N* = 13, elite male youth players 14.7 years, U15 players competing at regional and national competition	4 vs. 4 Possession	5 min bout duration, 2.5 min passive recovery	Physiological Variables: Session RPE ↑ (27.2%)
		4 vs. 4 Small goals		Physical Variables: Total player load ↑ (28.0%), relative player load ↑
Gaudino et al. (31)	*N* = 26, male professional players 26.0 years, competing in English Premier League & UEFA Champions League	5 vs. 5	Continuous format, variables were normalized per time	Physiological Variables: Total energy cost ↑, 20–35, 35–55, >55 W/kg ↑
		7 vs. 7		Physical Variables: TD↑ (5.81%), very-high speed running (19.8–25.2 km/h) ↓ (−44.8%), maxSpeed ↓ (−10,1%), max ACC ↓ (−9.17%) and DEC ↓ (−10.5%)
		10 vs. 10		
		Possession, GK		
Gonzalez-Rodenas et al. (39)	*N* = 20, elite male youth players 13.7 years, academy players from Spanish 1st division	4 vs. 4	4 min bout duration, 2 min passive recovery	Game format: HRmax 4 vs. 4 ↑ (4.54%) vs. 6 vs. 6
		6 vs. 6		
		Possession, GK		Physiological Variables: HRmax ↑ (10.8%)
Guard et al. (38)	*N* = 10, elite male youth players 18.0 years, participating in Scottish Premier and UEFA Youth League	5 vs. 5 Possession	Matched for format	Physiological Variables: Dur > 90% HRmax ↑ (39.7%), RPE ↑ (29.5%)
				Physical Variables: TD ↑ (11.0%), relative TD↑ (3.05%), Ave velocity ↑ (15.9%), #high-intensity efforts ↑ (100%), Dist 16.9–20.9 km/h ↑ (116%), Dist 14.0–16.9 km/h ↑ (117%), Dist 11.0–14.0 km/h ↑ (60.3%)
		5 vs. 5 GK		
Machado et al. (44)	*N* = 10, U15 non-elite male youth players 13.5 years, no experience in systematic game-based training	3 vs. 3	10 min bout duration, 10 min passive recovery	U15
				*3 vs. 3*
				Technical Variables: Dur ball possession ↑ (+>42.0%), players involved ↑ (+>18.9%), passes ↑ (+>96.7%), shots ↓ (-<61.0%), ball touches/Dur ↓ (-<32.9%), passes/Dur ↑ (+>34.6%), ball touches/players involved ↓ (-<19.6%), passes/players involved ↑ (+>63.6%), passes/ball touches ↑ (+>67.4%), goal/shots ↓ (-<88.9%)
		4 vs. 4		
				*4 vs. 4*
				Technical Variables: Dur ball possession ↑(+>43.4%), players involved ↑ (+>40.5%), ball touches ↑ (+>33.9%), passes ↑ (+>114%), shots ↓ (-<67.4%), ball touches/Dur ↓ (-<13.6%), passes/Dur ↑ (+>48.0%), passes/players involved ↑ (+>54.4%), passes/ball touches ↑ (+>57.5%), goal/shots ↓ (-<88.2%)
		Possession with GK, GK		
	*N* = 10, U17 non-elite male youth players 16.3 years, no experience in systematic game-based training			
				U17
				*3 vs. 3*
				Technical Variables: Dur ball possession ↑ (+>51.9%), players involved ↑(+>19.4%), ball touches ↑ (+>13.1%), passes ↑ (+>117.0%), shots ↓ (-<56.8%), ball touches/Dur ↓ (-<20.3%), passes/Dur ↑ (+>26.9%), passes/players involved ↑ (+>66.1%), passes/ball touches ↑ (+>50.0%)
				*4 vs. 4*
				Technical Variables: Players involved ↑ (+>22.6%), passes ↑ (+>64.5%), shots ↓ (-<69.8%), ball touches/Dur ↓ (-<26.9%), passes/Dur ↑ (+>32.0%), ball touches/players involved ↓ (-<26.7%), passes/player involved ↑ (+>39.1%), passes/ball touches ↑ (+>66.7%), goal/shots ↓ (-<91.3%)
Mallo et al. (28)	*N* = 10, male elite U19 (18.4 years), trained >4 times/week	3 vs. 3 Possession	5 min duration for each game	Physiological Variables: HRAve↑ (4.22%), Dur 86–95% HRmax↑, Dur 76–85% HRmax ↓
		3 vs. 3 + 2 GK		Physical Variables: TD ↑ (17.1%), Dist 13–18 km/h ↑, Dist > 18 km/h ↑
				Technical Variables: Contacts with the ball ↑ (69.2%), short Dist passes ↑ (124%)
Marasovic et al. (40)	*N* = 12, elite male youth players 18.44 years, participating in 1st Croatian Junior League	5 vs. 5	3–4 min bout duration, 3–4 min passive recovery	*5 vs. 5*
				Physiological Variables: Ave of 1st & 2nd set: HRmax ↓ (−2.69%), %HRmax ↓ (−2.63%), Dur 90–100% HRmax ↓ (66.1%)
		6 vs. 6		
				Physical Variables:
				Ave of 1st & 2nd set: TD ↓ (−21.4%) (for 2nd set), work rate/minute ↓ (−14.5%) (for 2nd set), Dist 14.76–18 km/h ↓ (−148%) (for 2nd set)
		Possession, GK		
				*6 vs. 6*
				Physiological Variables: Ave of 1st & 2nd set: HRmax ↓ (−2.18%), %HRmax ↓ (−2.57%)
				Physical Variables: Ave of 1st & 2nd set: <7.2 km/h ↑ (17.7%)
Pal'o et al. (35)	*N* = 18, male youth players 16.5 years, academy players from a 1st Slovakian league team	3 vs. 3 Possession	2 min bout duration, 1 min passive recovery	No statistical differences
		3 vs. 3 GK		
Rebelo et al. (34)	*N* = 10, male youth players 17.2 years, amateur level	5 vs. 5 Possession	10 min bout duration, 10 min passive recovery	Physiological Variables: Dur > 90% HRmax ↑, Dur < HRmax 80% ↓
		5 vs. 5 GK		Physical Variables: Dur jogging ↑, Dur striding ↑, Dur standing ↓, Dur walking ↓
				Technical Variables: Number of skill errors ↑
Segundo et al. 2021	*N* = 10, male youth players 12.45 years, member of regional U13 participating national championships	5 vs. 5 Possession	10 min bout duration, 5 min passive recovery	Physical Variables: Sprint Dist ↓ (−56.5%), #sprints ↓ (−38.9%), low-intensity running (3.0–8.0 km/h) ↑ (11.6%)
		5 vs. 5 GK		
				Tactical Variables: Width ↑ (20.1%)
Yilmaz et al. (30)	*N* = 24, male amateur players 21.0 years, trained >5 times/week	2 vs. 2	4 × 2 min duration for each game, 2 min passive recovery	*2 vs. 2*
		4 vs. 4		Physiological Variables: RPE ↑ (20.9%), mental fatigue VAS ↑ (18.4%), Enjoyment ↓ (−6.45%)
		Possession, mini-goal, GK		Technical Variables: #successful passes ↑
				*4 vs. 4*
				Physiological Variables: RPE ↑ (10,6%)

HR, heart rate; Ave, average; RPE, rating of perceived exertion; TD, total Distance; Dist, distance; Dur, duration; ACC, acceleration; DEC, deceleration; %, percentage; #, number, max, maximum; ↑, significant increase; ↓, significant decrease.

A total of 14 studies (221 male players) involving adult (five studies 90 participants) (28–32) and youth (nine studies 131 participants) (33–41) players, in different small-sided games (SSG) in different playing modes such as 2 vs. 2 (30), 3 vs. 3 (28, 32, 35, 37), 4 vs. 4 (29, 30, 36, 38, 39, 41), 5 vs. 5 (31–34, 40), 6 vs. 6 (39, 40), 7 vs. 7 (31, 32) and 10 vs. 10 (31) were explored.

### Physiological variables

Findings within the literature indicate that possession games seem to increase the physiological load in players compared to the scoring SSG. Variables associated with an increase in intensity such as mean (28, 32) and max heart rate (HR) (32, 39), duration >90% HRmax (34, 38), duration 86%–95% HRmax (28) as well as total energy cost (31) (in kj/kg) increased in the possession format. The players also subjectively felt greater exertion measured via internal load (duration x session ratio of perceived exertion RPE AU) (36) and rating of perceived exertion RPE (29, 30, 38). Furthermore, possession decreased variables indicative of lower average game intensity, such as time spent <80% HRmax (34), time spent 85%–89% (%HRpeak) (29), time spent 76%–85% HRmax (28). Interestingly, possession games also challenged players more mentally, as indicated by higher visual analog scale (VAS) scores in 2-a-side formats (30), which also coincided with lower enjoyment especially in comparison to regular goalkeeper participation (30). However, the duration in heart rate zones 76%–85% HRmax decreased in the possessional format (28).

There also seems to be an effect of number of players on physiological variables. An increase in player numbers appeared to diminish the dominant physiological effect of ball possession SSG. More precisely, the effect of RPE and mental fatigue decreased from a 2 vs. 2 game format to a 4 vs. 4 format (30). Furthermore, 3 vs. 3 and 5 vs. 5 possession games were observed to elicit higher HRmean and HRmax values compared to small-goal and goalkeeping with the same number of players (32) while there was no difference in physiological variables in a 7 vs. 7 anymore (32). Similarly, higher significant percentage maximum heart rate (%HRmax) was observed in 4 vs. 4 ball possession compared to goal scoring SSG, however, not in a 6 vs. 6 anymore (39). To conclude the section, it needs to be stated that the first set of two during scoring SSG identified significantly higher HRmax, %HRmax, duration 90%–100% HRmax compared to the possession format (40), which opposes the previously mentioned research for the 5 vs. 5 game format somehow. The %HRmax as well as the HRmax were also significantly different in the 6 vs. 6 format (40). Nevertheless, only the first set of two was significantly different, questioning the entire games, as the second set of possession game was not significantly different from the games including GK.

### Physical variables

Possession compared to scoring-base SSG have been shown to increase the physical demands placed on players during SSG. Indicators of greater physical involvement, such as total distance (28, 29, 31, 32, 38, 41, 42), distance relative to minutes played (29, 38), and average moving speed (29, 41) have been reported to be greater in possession games. Furthermore, greater distances in high(er) speed bands such as >18 km/h (28), 16.92–20.88 km/h (38), 14.04–16.92 km/h (38), 13–18 km/h (28, 32), 10.98–14.04 km/h (38), 7–12.9 km/h (29, 32), 3.6–14.3 (km/h) (41) were reported in possession SSG. Players also appear to experience greater mechanical load measured via acceleration (ACC) and deceleration (DEC). Possession games have been associated with greater total and relative player load (32, 36), total number of DEC (42), number of high-ACC (≥2.5 (m/s^2^) (29), as well as all metabolic parameters, including metabolic power, total high power output (>20 W/kg), and power across multiple bands (20–35, 35–55, and >55 W/kg) (31). The players also received less pause during these games and the work-to-rest ratio (32) was greater compared to scoring games. Unsurprisingly, even less intense physical metrics such as distances at speed 3–8 km/h (33), duration (34) and distance jogging (41), and duration of striding (34) were greater in possession games. Furthermore, variables indicative of lower physical strain, such as distances standing and walking [<3.6 km/h (28, 33, 41) and <7.2 km/h (28, 32)] were lower in possession games. However, it should be noted that opposing evidence is limited. Only the number (33) and distance sprinting (33) (in a 5 vs. 5), very high-speed running (19.8–25.2 km/h) (31), maximum speed (31), maximum ACC and DEC (31), and number of low-ACC (1.0–1.4 m/s^2^) (29) were observed to be significantly higher in scoring games.

End-of-spectrum variables, such as sprinting (>21 km/h), at the highest, and walking/standing (0–6.9 km/h) at the lowest, should be interpreted with caution, as they appear sensitive to changes in player numbers. In contrast, mid-range speed categories (7–12.9 and 13–17.9 km/h) maintain the rang order showing possession games significantly higher compared to scoring games (32). Indeed, relatively large games (9 vs. 9) seems to inherit greater individual positional responsibilities compared to task constraint, such as ball possession. Generally, the ball possession SSG were observed to hold higher physical load for certain variables [total distance, peak speed (km/h), and player load (AU)] in comparison with position games (43), while the latter inherited greater mechanical variables such as maximal ACC, maximal DEC, number of ACC > 3 m/s/s and number of DEC <3 m/s/s (43). Similarly to the findings for physiological variables, Marasovi et al. (40) displayed significant lower values for total distance, work rate, as well as distance covered 7.56–10.8 km/h in the first set of possession game, however, significant lower values in high-intensity (>16.2 km/h), distance >18 km/h, distance 14.76–17.99 km/h in the second set or possession game. It seems feasible to skeptically acknowledge the variables by Marasovi et al. (40) as values for distance >18 km/h and distance 14.76–18 km/h was reduced from the 1st to the 2nd series by 70% and 41% respectively.

### Technical variables

The literature revealed that playing possession games increased the technical involvement of players, especially concerning passes. Variables measuring technical activity such as total ball touches (28, 44), total number of passes (44), passes per player (44) and possession duration (44) are higher in possession compared to scoring games. Naturally with a greater number of total passes, the number of short (28, 33), and long distance passes (33) as well as the number of successful (30) and unsuccessful passes (in short distance) (28) and the number of skill errors (34) increases. Interestingly, during ball possession SSG, players used less touches on the ball to accomplish a pass (44) indicating more efficient ball handling and possibly even less dribbling. Furthermore, whilst not statistically significant, the number of passes, receives and turns were on average higher in the possession format (40). Nevertheless, ball touches per possession duration, ball touches per player, as well as the number goals/shots decreased (44). Interestingly, there appeared to be a possible effect of the number of players on technical variables during SSG. For example, a greater number of technical variables (number of successful and unsuccessful passes and interceptions) were significantly higher in possession vs. scoring games in a 2 vs. 2 format. While only one variable (successful passes) remained significant in a 4 vs. 4 format (30). The superior frequency of technical actions seems evident not only in possession vs. scoring games, but also with regards to playing on smaller pitch sizes (45–47). However, larg(er) pitch sizes created more opportunities to maintain ball possession, spent more time looking for the best solution to score a goal, and an inherited defensive line closer to the goal line (45). There seemed to be an effect of player number in combination with player age on technical variables (44). Most technical variables (duration of ball possession, ball touches, passes, ball touches/duration, ball touches/player involved, passes/ball touches) were greater in older players (U17) in the small format (3 vs. 3), while most of these variables (duration of ball possession, ball touches, passes, ball touches/player involved) in addition to others (passes/player involved) showed reverse significance (U15 > U17) with greater number of players (4 vs. 4) (44).

### Tactical variables

From the scarce scientific evidence regarding the effect of possession games, it seems that more players were involved for a longer period of time exploring greater space during possession games. Indicative variables such as players involved/duration (44), duration of ball possession (44), and the spatial exploration index (41) were significantly greater in possession compared to scoring during SSG. However, possession games also decreased the distance to nearest teammate and opponent (41) as well as passing decision-making (41). Interestingly, teams composed by younger players (U15) presented greater difficulties in maintaining ball possession in SSG, compared to U17 players (44).

## Conclusion

This narrative mini-review aimed to provide a first overview of the influence of ball possession compared to goal-scoring SSG in soccer. Ball possession is a specific task constraint implemented frequently by coaches during SSG. However, there are several consequences utilizing possession games with regards to physiological, physical, technical and tactical outcomes. Possession games increase the physiological load in players. Coaches can increase heart rate and the duration >90%HRmax for their players, who subjectively feel greater exertion in possession games. Therefore, this makes possession SSG particularly effective for aerobic conditioning, especially in small formats (2 vs. 2–4 vs. 4), whilst subjectively very tasking. However, as player numbers increase (≥6 vs. 6), physiological differences between possession and scoring formats diminish. Coaches should therefore prioritize smaller possession games when the goal is to overload cardiovascular systems, while larger formats may be used for physiologically less demanding sessions and positional-specific training.

Players will also experience greater physical strain whilst playing possession SSG. Greater total distance, distance per minute with greater average moving speed, more distance in high(er) speed bands (e.g., >18 km/h) and mechanical load through ACC and DEC can be expected with implementing possession games. These characteristics make possession SSG suitable for developing physical work capacity, particularly, considering higher-work-to-rest ratio. However, it seems that scoring games may better stimulate maximal sprinting and peak speed actions, suggesting both formats should be alternated depending on the targeted physical quality.

The present narrative mini-review indicates that technical execution (e.g., passing frequency, ball touches) can be increased through possession SSG. This makes them useful for coaches working with cohorts where the primary goal is to enhance technical skills by exposing players to constant technical involvement (e.g., individual sessions). However, the reduced number of shots and goal-related actions suggests that possession games should be complemented with scoring-based tasks when finishing and attacking outcomes are prioritized.

The limited evidence regarding the tactical consequences in SSG due to possession games indicate that greater number of players are involved for extended period of time exploring greater space, while decreasing distance to nearest opponent. From a practical perspective, possession SSG appear suited for training objectives related to collective organization in possession, particularly when the aim is to develop coordinated team behaviors. Additionally, emerging evidence suggests that possession SSG may encourage players to utilize a greater proportion of the available playing space. Coaches can exploit this by designing possession tasks that emphasize width and depth, as well as continuous off-ball movement and adjustment (e.g., during build-up phases).

The aforementioned should be interpreted cautiously given the limited and somehow heterogeneous evidence. More precisely, this narrative mini-review utilized all available scientific evidence regarding the effects of possession format SSG in comparison to goal scoring SSG involving any types of goals and their effect on physiological, physical, technical and tactical parameters disregarding various other task constraints such as the relative pitch size (48), number of players (32), and touch restrictions (49), that influenced the aforementioned parameters. In addition, the statements within each section (physiological, physical, technical, tactical) might be based on different populations (youth and adults) with various playing experience and skill level. However, due to the limited available research with regards to possession SSG in comparison to scoring-format, the available scientific information was pooled in this narrative mini-review. Consequently, more research using more format configurations (e.g., relative pitch size, number of players) in various populations (e.g., male, female, youth and adults) is required to provide more generalisable recommendations for coaches. Coaches should be cognizant that younger footballers might present difficulties maintaining possession during SSG. Nevertheless, in the light of these limitations, coaches can utilize possession SSG to influence the physiological, physical, and technical demands during SSG.
